# Fruit Smoothie Supplementation and Inflammatory Biomarkers in Individuals Receiving Opioid Agonist Therapy: A Multicenter Randomized Controlled Trial

**DOI:** 10.3390/nu18213623

**Published:** 2026-10-31

**Authors:** Lise M. Thomassen, Elaheh J. Arjmand, Karl T. Druckrey-Fiskaaen, Einar Furulund, Jørn Henrik Vold, Rune Blomhoff, Hege Berg Henriksen, Tesfaye Madebo, Jan T. Daltveit, Fatemeh Chalabianloo, Torgeir Gilje Lid, Lars T. Fadnes, for the ATLAS4LAR and FruktBAR study group

**Affiliations:** 1 Department of Global Public Health and Primary Care, University of Bergen, Årstadveien 17, 5009 Bergen, Norway; 2 Bergen Addiction Research, Department of Addiction Medicine, Haukeland University Hospital, 5021 Bergen, Norway; 3 Center for Alcohol and Drug Research, Stavanger University Hospital, 4068 Stavanger, Norway; 4 Oral Health Centre of Expertise, 4001 Stavanger, Norway; 5 Division of Psychiatry, Haukeland University Hospital, 5021 Bergen, Norway; 6 Department of Nutrition, University of Oslo, 0372 Oslo, Norway; 7 Department of Clinical Service, Division of Cancer Medicine, Oslo University Hospital, 0424 Oslo, Norway; 8 Norwegian Institute of Public Health Services, 0213 Oslo, Norway; 9 Department of Respiratory Medicine, Stavanger University Hospital, 4068 Stavanger, Norway; 10 Department of Clinical Science, University of Bergen, 5020 Bergen, Norway; 11 Norwegian Center for Agonist Treatment of Substance Use Disorders (NORCATS), Department of Addiction, Haukeland University Hospital, 5021 Bergen, Norway; 12 Faculty for Health Sciences, University of Stavanger, 4036 Stavanger, Norway

**Keywords:** CRP, cytokines, inflammation, nutrition, opioid agonist therapy, randomized controlled trial

## Abstract

*Background/Objectives:* People with opioid dependence often experience elevated inflammatory markers and poor dietary habits. Evidence from other populations suggests that higher fruit and vegetable intake may reduce inflammation. This multicenter randomized controlled trial investigates whether offering a daily supplementation of 250 mL of fruit smoothie for 16 weeks to people undergoing opioid agonist treatment (OAT) can reduce inflammation biomarkers. *Methods:* 319 individuals were randomized to receive either a daily supplement of 250 mL of fruit smoothie in addition to standard OAT or standard OAT alone over 16 weeks. The primary outcome was the log-transformed change in high-sensitivity C-reactive protein (hs-CRP). Secondary outcomes included changes in cytokines (IL-8, IL-1RA, and MCP-1) and leukocyte counts. *Results:* No between-group differences were observed in changes in hs-CRP levels (0.96 (0.77; 1.21) mg/L), leukocytes (0.99 (0.93; 1.05)10^9^/L), IL-8 (1.10 (0.84; 1.46) pg/mL), MCP-1 (0.94 (0.80; 1.11) pg/mL), and IL-1RA (1.06 (0.91; 1.23) pg/mL). High levels of inflammatory markers were observed at baseline in both the intervention group and the control group. *Conclusions:* After 16 weeks of offering fruit smoothie supplementation in patients receiving OAT, we did not detect a between-group reduction in inflammation markers such as hs-CRP, IL-8, MCP-1, IL-1RA, and leukocyte counts, under the achieved level of delivery and exposure. Nonetheless, persistently high levels of inflammatory markers were observed in this study.

## 1. Introduction

Opioid dependence (OD) is associated with several serious adverse health outcomes, including infectious diseases such as hepatitis C virus and human immunodeficiency virus, as well as an increased risk of overdose and death [1]. Chronic opioid use also has numerous nutritional consequences, placing this population at high risk of malnutrition [2]. Opioid agonist treatment (OAT) is the most effective treatment for managing OD [1,2]. However, despite receiving OAT, individuals with OD continue to experience a substantially higher burden of morbidity than the general population.

Among the various physiological processes affected by opioid use, chronic opioid exposure has been shown to modulate immune function, particularly inflammatory responses [3]. These effects may arise indirectly through bacterial metabolites associated with injecting substances or through direct interaction between opioid receptors expressed on immune cells [4]. The effects of OAT on inflammatory biomarkers have also been investigated, although findings remain inconsistent. Some studies have reported significant reductions in inflammatory biomarkers among individuals receiving methadone or buprenorphine [5], whereas others suggest that these medications may promote oxidative stress [6]. Although acute inflammation is an essential protective response that contributes to the restoration of physiological homeostasis, persistent or chronic inflammation is considered unfavorable. Prolonged inflammatory activity may disrupt normal physiological processes and has been linked to an increased risk of several chronic diseases, including type 2 diabetes, cardiovascular disease, and cancer [7].

Opioid use has been associated with unhealthy eating behaviors, potentially due to elevated dopamine levels that suppress appetite and reduce interest in food. This may lead to decreased overall food intake or a shift towards unhealthy dietary choices [2]. Previous studies have reported particularly low fruit and vegetable consumption among individuals receiving OAT [8,9]. A recent systematic review and meta-analysis found that higher fruit and vegetable intake was associated with lower levels of pro-inflammatory mediators and improvements in immune cell profiles across various diseases and health conditions [10]. Furthermore, studies involving individuals with depression have demonstrated the potential of dietary interventions to improve mental health outcomes [11,12], which have also been linked to inflammatory processes [13]. Intervention studies using antioxidant-rich foods have also shown a change in blood cell gene expression linked with cellular protection [14]. Taken together, these findings suggest that fruit smoothies may represent a promising dietary intervention to reduce chronic inflammation among individuals with OD. Therefore, this randomized controlled trial aims to investigate whether offering a daily supplementation of 250 mL fruit smoothie over 16 weeks reduces chronic inflammation in individuals receiving OAT, as measured by changes in inflammation biomarkers.

## 2. Materials and Methods

*Study design and setting:* This multicenter, individually randomized controlled trial was conducted at seven OAT clinics in Bergen and Stavanger, Norway, between April 2022 and October 2023. The study protocol has been published, and a detailed statistical analysis plan was developed prior to data handling [15]. The target population comprised individuals diagnosed with opioid dependence syndrome according to the International Statistical Classification of Diseases and Related Health Problems, 10th Revision (ICD-10), who were receiving OAT at the time of enrolment [16]. Written informed consent was obtained from all eligible participants before enrolment. The study protocol was approved by the South-Eastern Regional Ethical Committee (155386/REK-B, dated 23.09.2020/03.12.2021/05.04.2022). The results are reported in accordance with the Consolidated Standards of Reporting Trials (CONSORT) guidelines.

**Figure 1:**
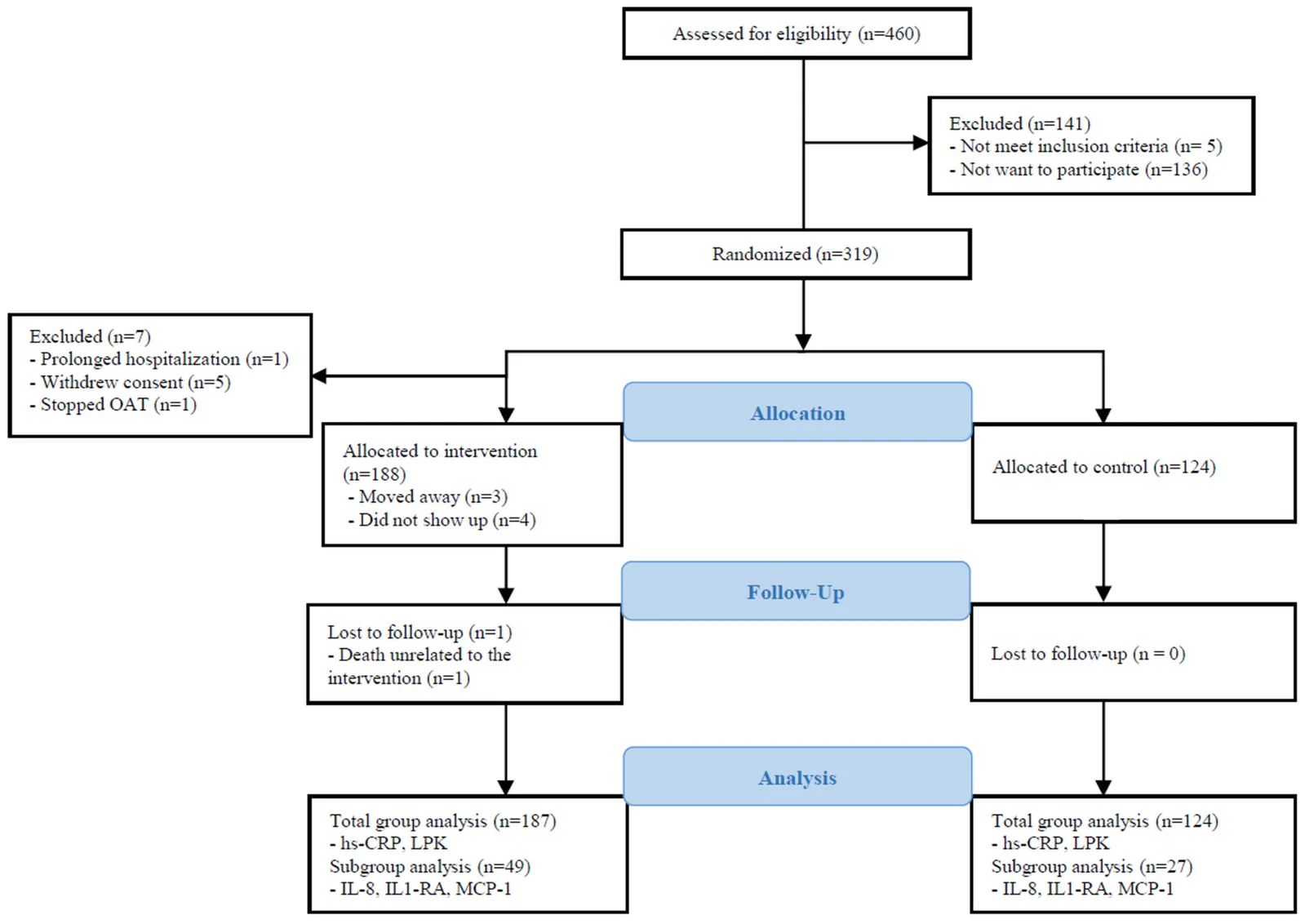
Flow chart for the patient recruitment and data selection process.

*Participants:* This study was conducted as part of the ATLAS4LAR project, which aims to improve the well-being of individuals receiving OAT, with particular focus on lifestyle factors [17,18,19,20]. Participants were recruited during annual health assessments at the participating clinics. The inclusion criteria were receiving OAT with weekly outpatient follow-up and reporting intake of less than five servings per day of fruits or vegetables. Exclusion criteria were known allergies or a history of anaphylactic reactions to fruits or vegetables, as well as poorly regulated type 1 or type 2 diabetes mellitus, defined according to national guidelines by an HbA1c level ≥ 54 mmol/mol [21]. A total of 460 individuals were assessed for eligibility, of whom 319 met the inclusion criteria and were randomized to either the intervention group (*n* = 195), receiving 250 mL fruit smoothie supplement, or the control group (*n* = 124), receiving standard care for 16 weeks (Figure 1).

Of the 195 participants allocated to the intervention group, seven withdrew before the baseline visit, and one was lost to follow-up. Consequently, 187 participants in the intervention group and 124 participants in the control group were included in the final analysis of high-sensitivity C-reactive protein (hs-CRP) and leukocyte count. A subgroup of participants was additionally randomized to provide dried blood spot samples for the assessment of interleukin 8 (IL-8), interleukin-1 receptor antagonist (IL-1RA) and monocyte chemoattractant protein-1 (MCP-1). This subgroup comprised 76 participants, of whom 49 were in the intervention group, and 27 were in the control group.

*Randomization and masking:* Eligible patients were randomized to either the intervention or control group using an allocation ratio of 5:3. This ratio was changed from the 1:1 allocation specified in the initial protocol following the identification of a coding error in the randomization algorithm, as described in the revised protocol [22]. Randomization was performed using the CheckWare data entry system (CheckWare, Trondheim, Norway), which automatically assigned concealed identification numbers to each participant. One researcher not involved in the data collection (LTF) and one randomization assistant had access to the randomization list. A subgroup of participants was randomized to dried blood spot testing with a 1:4 allocation ratio among trial participants.

*Blinding:* Although full blinding of the participants was not feasible in this trial, participants were not informed of the specific hypothesis or the nature of the intervention provided to the other group. The data analyst (JHV) remained blinded to group allocation until the analyses were completed. Study nurses were instructed not to document or disclose participants’ randomization status. Furthermore, participants were not informed about follow-up procedures in the other group.

*Sample size:* This paper reports secondary outcome measures from the FruktBAR study, for which the original sample size calculation was based on the primary outcome of the trial. Additional power calculation was performed for hs-CRP, the primary outcome measure in the present analysis, and one of the cytokines, IL-8. The study had 72% statistical power to detect a 40% relative reduction in hs-CRP levels compared with the baseline levels observed in the control group (8.5; SD = 11.6). For IL-8, the study had 51% power to detect a 50% relative reduction compared with the baseline levels observed in the control group (165; SD = 176).

*Intervention:* Participants in the intervention group received a daily 250 mL fruit smoothie supplement for 16 weeks. The smoothies comprised four different commercially available products from BAMA Gruppen AS (Bendit) in Norway, purchased through a regular supplier without any financial support or other contributions from the manufacturer. The nutritional composition of each smoothie type is presented in Table S1. Participants received seven bottles of smoothie each week during their daily visits to the outpatient clinic to collect their OAT medication and were instructed to consume one bottle per day. A selection of the four smoothie varieties was provided, and participants could choose products according to their personal preferences. Adherence to the intervention was assessed through weekly self-reports, in which participants reported the number of smoothies consumed during the preceding week.

*Control:* Participants in the control group received standard follow-up care at their respective OAT clinics and did not receive fruit smoothie supplementation.

*OAT clinic follow-up:* In the OAT clinics, patients receive methadone, buprenorphine, or morphine sulphate as part of their OAT, either as directly observed therapy or as take-home doses. The number of take-home doses of OAT per week ranges from one to six (determined based on factors such as concurrent use of illicit substances, mental and physical comorbidities, and treatment adherence). During the COVID-19 pandemic, medication was usually delivered to patients’ homes by OAT staff. Each patient is assigned a primary therapist (typically a nurse or social worker) and is offered at least one health assessment conducted by a physician and nurse. The OAT clinics maintain close collaboration with patients’ primary care physicians, municipal social workers, and other municipal and peer organizations for persons with substance dependence. Additional information on OAT clinics is provided in Text S1.

*Outcomes and assessments:* The main outcome of the study was the change in hs-CRP levels from baseline to the end of the 16-week intervention. Secondary outcomes included changes in IL-8, IL-1RA, MCP-1, and leukocyte count. Non-fasting venous blood samples were collected from all study participants at baseline and at the end of the intervention to measure hs-CRP and leukocyte count. Samples from participants in Bergen were analyzed at the Department of Medical Biochemistry and Pharmacology, Haukeland University Hospital, while those from Stavanger were analyzed at the Department of Medical Biochemistry, Stavanger University Hospital. Both laboratories are certified according to NS-EN-ISO 15189:2022 standard [23]. In addition, non-fasting dried blood spot samples were collected from a subgroup of participants (*n* = 76) to assess IL-8, IL-1RA, and MCP-1 levels. A detailed description of the dried blood spot sampling and laboratory analysis is provided in Text S2.

*Statistical analysis:* All statistical analyses were conducted using Stata/SE17 (StataCorp, College Station, TX, USA), while data visualization was performed in R (version 2024.04.02+764) using the Tidyverse package (version 2.0.0). The significance threshold was *p* < 0.05. Modified intention-to-treat analysis was performed using data from all randomized participants, while per-protocol analysis included patients who received at least 75% of the offered fruit smoothies. Participants without a baseline blood sample were excluded from the analysis. Missing data at the end of treatment or blood samples collected ≥28 days after the end of treatment were handled using baseline values carried forward. For each log-transformed outcome variable, linear mixed models with restricted maximum likelihood estimation were performed to assess the difference between groups in change from baseline to end of treatment. The mixed-effects model was fitted to the repeated baseline and end-of-treatment (EOT) measurements. The outcome variable was expressed as log-transformed change relative to baseline, but observations at both time points were included in the model. Fixed effects included time, treatment group, and the time-by-treatment interaction, while participant ID was included as a random intercept to account for within-subject correlation arising from repeated measurements. Variables were log-transformed for statistical analysis. Baseline and end-of-treatment values are presented as geometric means (geometric standard deviations), while between-group comparisons are presented as geometric mean ratios with 95% confidence intervals. All results are back-transformed to the original scale for presentation.

## 3. Results

### 3.1. Baseline Demographics

In the intervention group, the mean age was 47 (SD 11) years, and the mean body mass index was 26 (SD 6) kg/m^2^ (Table 1). The majority of patients were male (70%), and 55% were receiving buprenorphine as their OAT medication. Many of the patients reported substance use within the last 30 days, including opioids (23%), alcohol (50%), stimulants (33%), benzodiazepines (47%), and cannabis (60%). Additionally, 12% reported injecting substances at least once per week. Most participants had an intake of fruits and vegetables below two servings per day (70%), and only 4% reported a high intake, defined as 4 or more servings per day. The majority of participants reported having high psychological distress (70%). A total of 54% had less than ten years of formal education, while 96% reported income sources other than paid work. Baseline demographics of the control group were similar. Baseline demographics for the subgroup of participants who provided dried blood spot samples are presented in Table S2.

### 3.2. Inflammation Biomarkers

**Figure 2:**
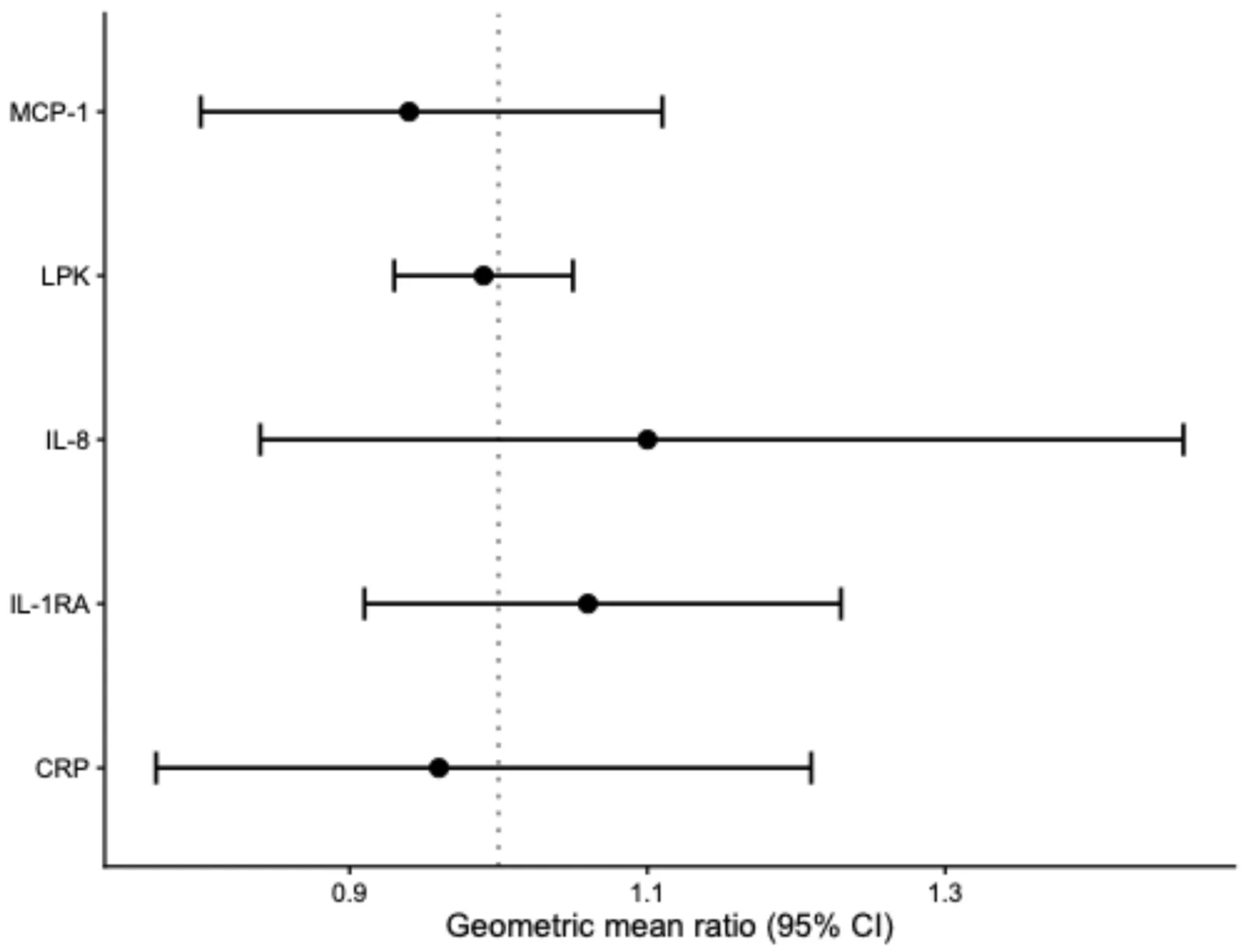
The change difference across groups and corresponding 95% confidence intervals for inflammation biomarkers. CRP: high-sensitivity C-reactive protein; IL-8: interleukin-8; IL-1RA: interleukin-1 receptor antagonist; MCP-1: monocyte chemoattractant protein-1; and LPK: leukocyte count.

Following 16 weeks of offering fruit smoothie supplementation, there were no differences in inflammatory biomarkers when comparing change from baseline to end of treatment between the intervention and the control group: hs-CRP levels (0.96 (0.77; 1.21) mg/L), leukocytes (0.99 (0.93; 1.05)10^9^/L), IL-8 (1.10 (0.84; 1.46) pg/mL), MCP-1 (0.94 (0.80; 1.11) pg/mL) and IL-1RA (1.06 (0.91; 1.23) pg/mL) (Figure 2 and Table 2). High levels of inflammatory markers were observed at baseline in both the intervention group and the control group.

Similar results were seen in the per-protocol analyses for patients receiving more than 75% of fruit smoothies (Table S3). The percentage of smoothie bottles delivered to the intervention group was 88% (Figure S1), and the mean number of smoothies consumed per week was 3.9 (SD: 1.5) among those who received 75% or more of the offered smoothie bottles (Figure S2).

## 4. Discussion

This randomized controlled multicenter study assessed the effect of offering daily supplementation with 250 mL fruit smoothie for 16 weeks on inflammatory biomarkers among patients receiving OAT. No statistically significant between-group differences in reductions in inflammatory markers were observed. However, inflammatory marker concentrations remained notably elevated throughout the study. To our knowledge, this is the first trial to investigate the effects of offering fruit smoothie supplementation on inflammatory markers in individuals receiving OAT. A previous study has shown that both buprenorphine and methadone may induce oxidative stress, with patients receiving these medications exhibiting significantly higher hs-CRP levels compared to healthy controls [6]. Conversely, a systematic review has challenged these findings, suggesting that maintenance therapy with buprenorphine and methadone may reduce chronic inflammation and oxidative stress [5]. Consistent with previous research reporting dysregulation of inflammatory biomarkers among people with opioid use disorder, our findings suggest that elevated inflammatory markers may persist among individuals receiving OAT, highlighting the need for further research into the inflammatory burden in this population [5]. Interestingly, CRP and LPK levels showed slight increases from baseline to end of treatment in both the intervention and control groups. However, the between-group differences in change were not statistically significant, suggesting that the intervention did not produce a demonstrably different effect on these markers compared with the control condition. The observed increases may reflect temporal variation or other factors unrelated to the intervention, including infections such as COVID-19, although the underlying reasons cannot be established from these findings alone.

Fruit and vegetable consumption has been associated with reduced inflammatory markers in both observational studies and clinical trials across populations with various diseases and conditions [10]. Although several compounds found in fruits and vegetables exhibit antioxidant properties that may contribute to their anti-inflammatory effects by mitigating oxidative stress and modulating inflammatory pathways, the specific compounds responsible and the underlying mechanisms remain incompletely understood. Nevertheless, various compounds have been identified as contributing to the antioxidant capacity of fruits and vegetables, potentially influencing pathways in oxidative stress and inflammation [24,25,26]. In the Oslo Antioxidant study, an 8-week intervention with the provision of kiwi or antioxidant-rich meals among people smoking tobacco resulted in changes in gene expression related to cellular protective mechanisms [14]. However, a study involving patients with chronic obstructive pulmonary disease found that a 12-week increase in fruit and vegetable intake did not significantly alter markers of airway or systemic oxidative stress and inflammation [27]. In the FruktBAR study, no significant differences in carotenoid levels were observed between the intervention and control group. However, carotenoids may not be an ideal biomarker of intake for the specific fruits used in the smoothies in our trial. It is also important to note that isolating the specific effects of dietary interventions on inflammation is inherently challenging, as inflammation is a multifactorial process influenced by a range of factors beyond diet, including stress, sleep, physical activity, infections, and environmental exposures [7,28].

To enable comparison between cytokine concentrations measured in dried blood spots in our study and those measured in plasma in previous studies, we applied a correction factor of 2 to the cytokine concentrations, as recommended by previous research [29]. Following this adjustment, IL-1RA levels in our study were nearly three times higher than those reported in a study linking opium use to elevated IL-1RA levels in people with coronary artery disease [30]. Similarly, IL-8 levels in our study were approximately fourteen times higher than those reported by Wang et al. (2023), who investigated associations between inflammatory cytokines and substance use in patients with opioid and stimulant use disorders [31]. The agreement between cytokine concentrations measured in dried blood spots and plasma samples has been examined by McDade and colleagues, who found high agreement for most cytokines, with the notable exception of IL-8. In their study, IL-8 concentrations measured in dried blood spot samples were substantially higher than those in matched plasma samples, suggesting a potential measurement bias [32]. One possible explanation is that DBS samples contain red blood cells that have high concentrations of cytokines such as IL-8 [33], and we wonder if this could explain the high values of inflammation markers measured using dried blood spots in our trial. In addition, the prolonged storage of non-filtered blood may have contributed to the elevated IL-1RA levels [34].

The current trial has several strengths, including its design and the recruitment of individuals who are often difficult to reach through traditional healthcare settings. In addition, the use of dried blood spots to measure inflammatory markers, such as cytokines, offers several advantages, including minimally invasive sampling and ease of collection [29,35,36,37]. However, there are also limitations to this trial. First, only a subgroup of 76 participants was allocated to dried blood spot sampling, providing data on IL-8, MCP-1, and IL-1RA. This reduced the statistical power to detect between-group differences in these outcomes. Second, blood samples were not consistently collected on the exact start and end dates of the intervention. In some cases, end-of-treatment samples were collected after completion of the intervention, potentially introducing variability in biomarker levels unrelated to the intervention itself. Third, many of the variables collected in this study, including fruit and vegetable intake, substance use, and living situation, were self-reported. This increased the risk of recall bias and may compromise data accuracy. Fourth, the small volume of blood collected by dried blood spot sampling, combined with the low concentration of cytokines in capillary blood, may have contributed to a high proportion of cytokine measurements falling below the lower limit of quantification. This limitation reduced the number of inflammatory markers available for statistical analysis and may have affected the study’s ability to detect changes. The statistical power to detect relevant changes for the cytokines was 51% and slightly below what is expected for primary outcomes, but the analytic power for C-reactive protein was 72%, which can be considered satisfactory. Finally, both qualitative data and some of the fruit smoothie consumption data suggest possible intervention leakage, whereby participants in the intervention group may have shared fruit smoothies with participants in the control group [19]. This may have reduced the ability to detect the effects of the intervention.

## 5. Conclusions

This study showed that a 16-week fruit smoothie supplementation intervention in patients receiving OAT did not detect a between-group reduction in inflammation markers such as hs-CRP, IL-8, MCP-1, IL-1RA, and leukocyte counts for people receiving OAT under the achieved level of delivery and exposure. However, for the cytokines, this must be interpreted with caution due to the insufficient sample size. Nevertheless, inflammatory marker concentrations remained elevated throughout the study period. Self-reported consumption data suggest suboptimal consumption of the fruit smoothies, even though most patients received nearly all of the planned fruit smoothies. These findings highlight the challenges of addressing inflammation in the OAT population and suggest that future studies should explore other interventions targeting unhealthy eating that may also modulate chronic inflammation in individuals receiving OAT.

## Figures and Tables

**Table 1:** Baseline demographics.

	Control (*n* = 124)	Intervention (*n* = 187)
Sex, Males/females, *n* (%)	87/37 (70/30)	131/56 (70/30)
Age, mean (SD)	48 (11)	47 (11)
BMI, mean (SD)	26 (6)	26 (6)
OAT, *n* (%)		
Methadone and morphine sulphate	59 (48)	85 (46)
Buprenorphine	65 (52)	102 (55)
Any weekly use of substances in the last 30 days, *n* (%)		
Illicit opioids, *n* (%)	22 (18)	42 (23)
Alcohol, *n* (%)	77 (62)	93 (50)
Amphetamines and cocaine, *n* (%)	42 (34)	61 (33)
Benzodiazepines, *n* (%)	62 (50)	86 (47)
Cannabis, *n* (%)	89 (72)	111 (60)
Injecting substances, *n* (%)	11 (9)	21 (12)
Fruit/vegetables intake, *n* (%)		
Low (<2 servings per day)	91 (73)	131 (70)
Moderate (≥2 & <4 servings per day)	25 (20)	48 (26)
High (≥4 servings per day)	8 (7)	8 (4)
High psychological distress, *n* (%)	86 (69)	130 (70)
Education, *n* (%)		
<10 years	60 (50)	98 (54)
≥10 years	61 (50)	82 (46)
Living in an owned or rented home, *n* (%)	119 (96)	177 (95)
Income, *n* (%)		
Other income *	120 (97)	185 (99.5)
Paid work	4 (3)	1 (0.5)

* Other income: unemployment benefits, sickness benefits, disability benefits, social benefits, savings or inheritance, student loan/grants/scholarships, or supported by others.

**Table 2:** Results from the modified intention-to-treat analysis showing the geometric mean (geometric standard deviation (%) at baseline and end of treatment (EOT) for the intervention and control groups, in addition to the change-difference across groups after 16 weeks of the intervention period. The two groups were compared using linear mixed models.

ITT	Intervention		Control		Geometric Mean Ratio Between Groups (95%CI)
	Geometric Mean (Geometric Standard Deviation (%))				
	**Baseline**	EOT	Baseline	EOT	
Hs-CRP (mg/L)	3.5 (±116%)	4.0 (±125%)	3.9 (±128%)	4.6 (±129%)	0.96 (0.77; 1.21)
IL-8 (pg/mL)	109 (±68%)	113 (±67%)	127 (±70%)	119 (±97%)	1.10 (0.84; 1.46)
IL-1RA (pg/mL)	2768 (±56%)	2775 (±54%)	2907 (±55%)	2753 (±57%)	1.06 (0.91; 1.23)
MCP-1 (pg/mL)	284 (±57%)	279 (±57%)	323 (±61%)	337 (±57%)	0.94 (0.80; 1.11)
LPK (10^9^/L)	6.9 (±51%)	7.2 (±54%)	7.2 (±50%)	7.6 (±51%)	0.99 (0.93; 1.05)

n_CRP,LPK_ = 311; n_IL-8, MCP-1, IL-1RA_= 76. CI: confidence interval; Hs-CRP: high-sensitivity C-reactive protein; IL-8: interleukin 8; IL-1RA: interleukin-1 receptor antagonist; MCP-1: monocyte chemoattractant protein-1; and LPK: leukocyte count.

## Data Availability

Data will be provided upon reasonable request.

## Supplementary Materials

The following supporting information can be downloaded at https://www.mdpi.com/article/10.3390/nu18213623/s1. Table S1: Nutrient content per 250ml of the BAMA fruit smoothies; Text S1: Description of OAT clinics; Text S2: Description of the handling and analysis of Dried Blood Spots; Text S3: Defining cut-off values for hs-CRP and leukocyte count; Table S2: Baseline demographics for the subgroup analysis of dried blood spots; Table S3: Results from the per protocol analysis showing; Figure S1: Percentage of frequency of delivery of smoothies; Figure S2: Average number of smoothie bottles consumed per week.

## Abbreviations

The following abbreviations are used in this manuscript:

CONSORT	Consolidated Standards of Reporting Trials guidelines
hs-CRP	high-sensitivity C-reactive protein
IL8	interleukin
IL-IRA	interleukin-1 receptor antagonist
ICD-10	International Statistical Classification of Diseases and Related Health Problems, 10th Revision
MCP-1	monocyte chemoattractant protein-1
OAT	opioid agonist treatment
OD	opioid dependence
SD	standard deviation
