# Supplementary material for: Fruit Smoothie Supplementation and Inflammatory Biomarkers in Individuals Receiving Opioid Agonist Therapy: A Multicenter Randomized Controlled Trial
Source: Nutrients. 2026 Oct 31;18(21):3623. doi: 10.3390/nu18213623 (PMC13764861; doi:10.3390/nu18213623)
Supplement: Supplementary file 1 [file nutrients-18-03623-s001.zip › nutrients-4589582-supplementary.pdf]

Supplementary Materials

Table S1: Nutrient content per 250 ml of the BAMA fruit smoothies.

	Mango and passion fruit	Pineapple and coconut	Blueberries and apple	Pineapple and mango
Energy (kcal)	125	160	133	118
Fat (g)	0.5	4.5	0.25	0.25
Saturated fatty acids (g)	0.25	4.3	0	0
Carbohydrates (g)	28	28	30	26
Protein (g)	1.25	1.5	1	1
Vitamin A (μg-RE)	22.5	2.5	5	17.5
Vitamin E (mg-ATE)	0.5	0.25	0.75	0.5
Thiamine (mg)	0.15	0.15	0.1	0.2
Riboflavin (mg)	0.3	0.13	0.3	0.15
Niacin (mg)	1.25	0.75	0.5	1
Pyridoxine (mg)	0.28	0.3	0.18	0.15
Folate (μg)	25	35	15	35
Ascorbic Acid (mg)	27.5	20	25	35

Text S1: Description of OAT clinics

The Department of Addiction Medicine at Haukeland University Hospital in Bergen and the Department of substance abuse and addiction treatment at Stavanger University Hospital in Stavanger have implemented an integrated treatment and care model for patients enrolled in OAT. These clinics operate with multidisciplinary teams consisting of medical consultants in addiction medicine, nurses, social workers, and in many cases, psychologists.

Text S2: Description of the handling and analysis of Dried Blood Spots

Dried Blood Spots was drawn either by sampling capillary blood using a lancet to prick the finger or by drawing a venous blood sample in a 3ml ethylenediaminetetraacetic acid tube and a DIFF-SAFE blood dispenser to the top of the tube. Droplets of blood was transferred to the paper that were set to dry in 2-4 hours in a dark room. The dried blood spot samples were stored in the fridge at -20 degrees. After study completion all dried blood spots were shipped frozen to the laboratory of Vitas Analytical Services (Oslo, Norway) for the measurement of inflammatory markers. Two 3.1 mm punches from the dried blood spots were eluted in kit diluent for 2 hours at 700rpm in room temperature. The inflammatory markers IL-8, IL-1RA and MCP-1 were measured on a MESO® QuickPlex SQ 120 Multiplex Imager using the custom U-PLEX Biomarker Group 1 Multiplex assay from Meso Scale Discovery, Rockville, Maryland, USA.

Text S3: Defining cut-off values for hs-CRP and leukocyte count

To lower the risk to have larger infections influence our results hs-CRP values > 60 mg/L at end of treatment were replaced with baseline value and leukocyte counts >12x10^9/L were set to 12. Leucocyte count <4x10^9/L were set to 4. Patients with missing baseline value or hs-CRP>60 mg/L at baseline were excluded from the analysis.

Table S2: Baseline demographics for the subgroup analysis of dried blood spots

	Control (N=27)	Intervention (N=49)
Sex, n (%)		
Male	19 (70)	32 (65)
Age, mean (SD)	46 (11)	47 (11)
BMI, mean (SD)	26 (6)	26 (7)
OAT treatment, n (%)		
Methadone and morphine sulphate	11 (41)	23 (47)
Buprenorphine	16 (59)	26 (53)
Living in owned or rented home, n (%)	27 (100%)	46 (96%)
Any weekly use of substances last 30 days, n (%)		
Illicit opioids, n (%)	6 (22)	11 (22)
Alcohol, n (%)	23 (85)	23 (47)
Amphetamines and cocaine, n (%)	12 (44)	19 (39)
Benzodiazepines, n (%)	15 (56)	26 (53)
Cannabis, n (%)	18 (67)	33 (67)
Injecting substances, n (%)	0 (0)	2 (4)
Fruit/vegetables intake, n (%)		
Low (< 2 servings per day)	19 (70)	35 (71)
Moderate (≥ 2 & <4 servings per day)	7 (26)	13 (27)
High (≥ 4 servings per day)	1 (4)	1 (2)
High psychological distress, n (%)	18 (67)	37 (76)
Education, n (%)		
<10 years	9 (35)	18 (42)
≥10 years	17 (65)	25 (58)
Income, n (%)		
Other income	25 (92)	48 (100)
Paid work	2 (7)	0 (0)

Table S3: *Results from the per protocol analysis showing the geometric mean (geometric standard deviation (%)) at baseline and end of treatment (EOT) for the intervention- and control group, in addition to the change-difference across groups after 16-weeks of intervention period. The two groups were compared using linear mixed models.*

**PP**	**Intervention**		**Control**		**Change difference across groups (GMR*, 95% CI)**
	Geometric mean (geometric standard deviation (%))				
	**Baseline**	**EOT**	**Baseline**	**EOT**	
Hs-CRP (mg/L)	3.3 (±115%)	3.9 (±128%)	3.9 (±128%)	4.6 (±129%)	0.96 (0.76; 1.21)
IL-8 (pg/ml)	111 (±61%)	113 (±57%)	127 (±70%)	119 (±97%)	1.11 (0.84; 1.46)
IL-1RA (pg/ml)	2801 (±57%)	2821 (±54%)	2907 (±55%)	2753 (±57%)	1.06 (0.90; 1.23)
MCP-1 (pg/ml)	288 (±58%)	276 (±58%)	323(±61%)	337(±57%)	0.94 (0.79; 1.11)
LPK (10^9/L)	6.9 (±52%)	7.0 (±56%)	7.2 (±50%)	7.6 (±51%)	1.00 (0.95; 1.05)

n for CRP,LPK = 247; n for IL-8, MCP-1, IL-1RA= 63. CI: confidence interval; Hs-CRP: high sensitive C-reactive protein; IL-8: interleukin 8, IL-1RA: interleukin-1 receptor antagonist, MCP-1: monocyte chemoattractant protein-1 and LPK: leukocyte count.

Figure S1: Percentage of frequency of delivery of smoothies

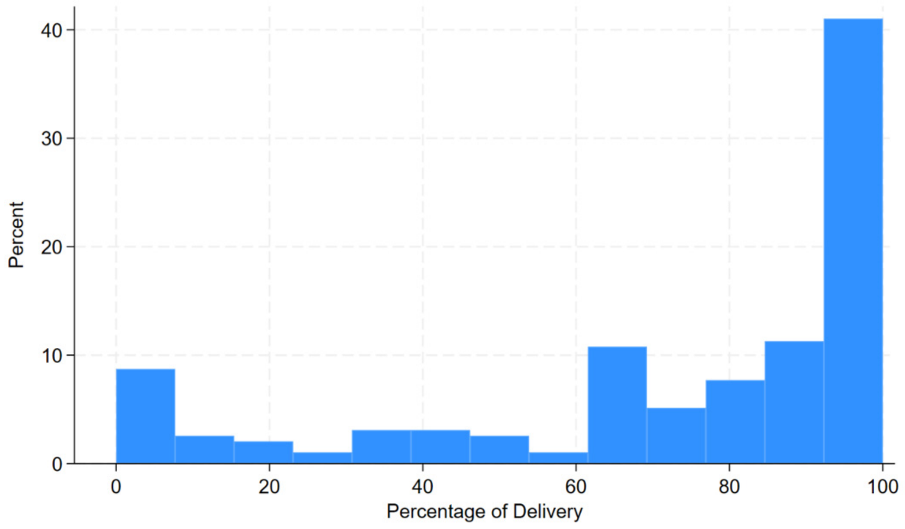

Figure S2: Average number of smoothie bottles consumed per week.

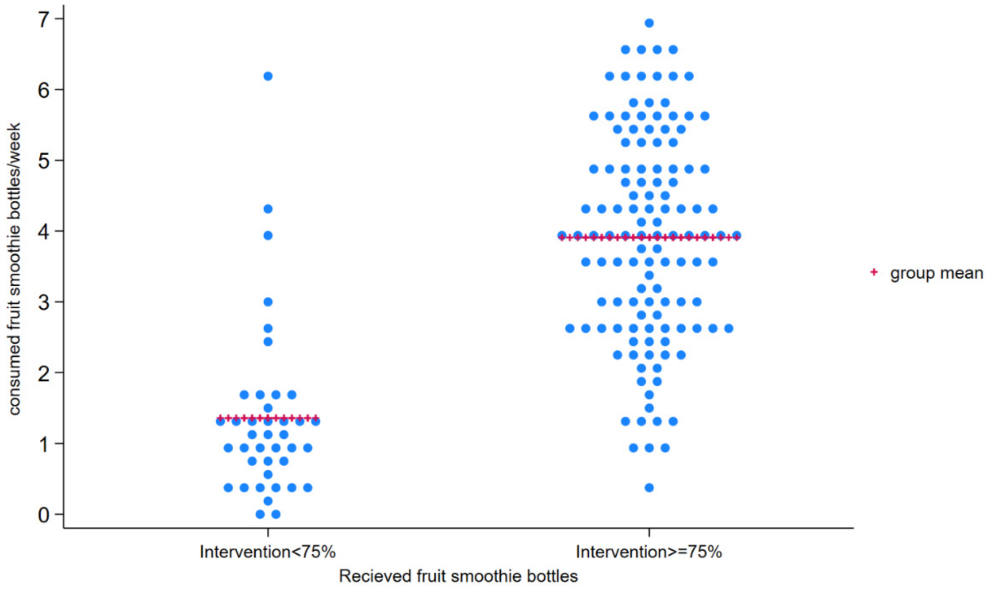

Each blue dot represents one participant in the intervention group. The red line represents the mean in each group (Received <75% of the offered smoothies, and >= 75%).
